# Orthogonally and linearly polarized green emission from a semipolar InGaN based microcavity

**DOI:** 10.1515/nanoph-2023-0647

**Published:** 2023-12-18

**Authors:** Wei Ou, Yang Mei, Hao Long, Yukun Wang, Tao Yang, Yanhui Chen, Leiying Ying, Zhongming Zheng, Baoping Zhang

**Affiliations:** The School of Electronic Science and Engineering, Xiamen University, Xiamen 361005, China; College of Photonics and Electronics, Minnan Science and Technology University, Quanzhou 362332, China

**Keywords:** orthogonal and linear polarization, semipolar InGaN, microcavity

## Abstract

Polarized light has promising applications in biological inspections, displays, and precise measurements. Direct emission of polarized light from a semiconductor device is highly desired in order to reduce the size and energy-consumption of the whole system. In this study, we demonstrate a semipolar GaN-based microcavity light-emitting diode (MCLED) that could simultaneously produce green light with perpendicular and parallel polarizations to the c*-axis. Orthogonally polarized emission with a narrow linewidth (∼0.2 nm) arises from the valence band splitting and birefringent nature of the semipolar GaN material, as well as the mode selection of the resonant cavity. By modulating the cavity length, the device is capable of switching between single- and multi-mode emission spectra. We believe that the approach of employing a cavity structure and semipolar GaN can be extended to produce orthogonally and linearly polarized blue, red, and violet light by adjusting the material compositions.

## 1 Introduction

Polarized light sources play an important role in various daily applications [1–3] and basic scientific research. Utilizing polarized light as automotive lighting is able to prevent glare and reduce driving risks [1]. In biological observations, polarized light sources could help to obtain deeper information from inside biological tissues [2, 4]. A polarized white light source is also important for liquid crystal displays (LCDs) [5] and three-dimensional (3D) imaging [6, 7]. Additionally, in visible light communication (VLC), polarization division multiplexing shows obvious advantages [8, 9]. Compared with the normal-single linearly polarized light sources, orthogonally polarized light sources are capable of producing optical radiation with two orthogonally linearly polarized electric fields simultaneously and in many specific applications, such as generating different vector beams [10], gas detection [11], optical polarization encoding [12, 13], and nonlinear optics [14–16]. However, it is not easy to make a single light source with orthogonal linear polarization. Since the most common spontaneous emission light sources, such as light-emitting diodes (LEDs) and fluorescent lamps, do not exhibit polarization characteristics, linearly polarized light can only be obtained by using a polarizer to filter out photons whose vibration direction is not parallel to a specific slit direction [17, 18]. Orthogonal linearly polarized light can be produced by combining two separate light sources and two polarizers with orthogonal slit directions. This approach inevitably reduces the optical power of the light source because most of the photons are filtered by the polarizers [19, 20]. Orthogonal linearly polarized light emission is also achievable by employing two laser sources with orthogonal polar direction, which takes advantage of the inherent linear polarization properties. In addition to the laser itself, the exploitation of laser imaging and display is often hampered by the speckle effect that results from the coherent nature of laser light [21]. Two independent light sources are necessary for the above-mentioned approaches to realize orthogonally polarized emission. However, this will lead to enhance the size, power consumption, and cost of the light source, which hinders their practical application, particularly for portable application scenarios.

In contrast, birefringent crystals exhibit noticeable potential in simplifying the system. When unpolarized light passes through a birefringent crystal, the direction of vibration of the photons alters from isotropic to anisotropic, making it possible to produce light with orthogonal linear polarization [22–24]. Birefringence was initially discovered in 1669 by Rasmus Bartholin and was not well explained until the nineteenth century by Augustin-Jean Fresnel when light was recognized as a polarized electromagnetic wave. Currently, birefringence has been employed to produce and control polarized light [23, 24]. As a good example, the Zeeman laser is a conventional dual-frequency orthogonal laser consisting of a helium–neon laser, a birefringent crystal, and a special magnetic field [25]. However, many birefringent materials could hardly emit photons by themselves, such as orthorhombic crystals [26, 27], three-dimensional (3D) chiral metamaterials, and two-dimensional (2D) chiral metasurfaces [28]. Therefore, new materials and devices should be urgently developed to directly produce orthogonally polarized light.

Wurtzite GaN is a type of semiconductor material with superior optoelectronic properties. By doping with indium (In) or aluminum (Al), the emission wavelength of the material could be broadly tuned to cover the deep ultraviolet to the near-infrared [29–31]. For semipolar InGaN/GaN quantum wells, the emission exhibits polarization parallel to c* (E//c*) and perpendicular to c* (E⊥c*) due to the splitting of the valence band, where c* represents the projection of the **c**-axis **[0001]** on the plane [32–35]. In addition, since the wurtzite crystal is composed of two closely packed hexagonal sublattices, AlN, InN, GaN, and their ternary alloys exhibit biaxial refraction with super direction, along the **c**-axis or c*-axis [36]. Currently, the utilization of polarized InGaN LED light has shown significant advantages in polarization division multiplexing [37]. By combining polarization division multiplexing with wavelength division multiplexing, the data transmission rate could be appropriately enhanced in visible light communication (VLC) [38]. However, the wide spectra of conventional semipolar LEDs is prone to significant crosstalk, thereby compromising the quality of data transmission in wavelength division multiplexing [39, 40]. Consequently, achieving narrow and separated spectra for semipolar GaN devices represents a vital endeavor within the context of wavelength division multiplexing. Moreover, narrow polarized spectra with high color purity hold paramount importance for 3D displays [41].

In this study, we fabricate (20 $\bar{2}$ 1) semipolar InGaN/GaN-based micro-cavity light-emitting diodes, which are able to simultaneously emit a set of orthogonally polarized light along [10 $\bar{1}\bar{4}$ ] (//c*) and [1 $\bar{2}$ 10] (⊥c*) by a single device. Due to the mode selection of the resonant cavity, the spectral width is compressed, thus enabling the complete spectral separation of light with two distinct polarization directions. The line polarization degree of the device is close to 100 %. The full width at half maximum (FWHM) of the single longitudinal mode is about 0.2 nm, similar to the spectral width of conventional laser sources. In imaging applications, the MCLED light emission is incoherent and could avoid speckle effect compared to laser sources. Additionally, the spectral mode distribution of the device could be suitably altered by adjusting the cavity length of the resonant cavity to meet various application requirements.

## 2 Results

**Figure 1:**
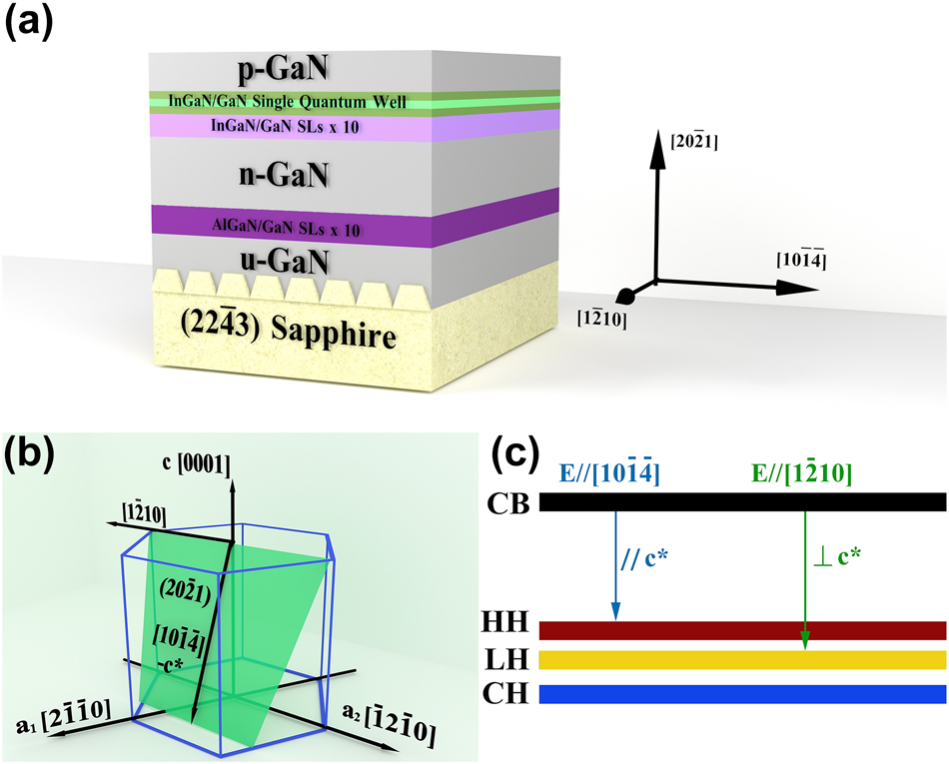
Schematic representation of: (a) the perspective cross-sectional view of the wafer structure; (b) (20 $\bar{2}$ 1) GaN in the wurtzite crystal structure; (c) band-edge energy of semipolar InGaN.

Figure 1(a) illustrates the structure of the epi-wafer. The epitaxial process of (20 $\bar{2}$ 1) semipolar GaN on a patterned sapphire (22 $\bar{4}$ 3) substrate (PSS) was performed via a low-pressure metalorganic chemical vapor deposition (MOCVD) system. The direction of the grooves and terraces of the sapphire substrate is parallel to [1 $\bar{2}$ 10]. From bottom to top, the epi-wafer consists of undoped GaN, 10 pairs of AlGaN/GaN superlattice, *n*-type GaN layer, 10 pairs of InGaN/GaN superlattice, a single InGaN/GaN quantum well, AlGaN electronic barrier layer (EBL), and p-type GaN layer. A schematic representation of the near-hexagonal crystal structure of GaN is provided in Figure 1(b). After defining the crystal plane and labelling the crystal direction, [1 $\bar{2}$ 10] and the unit vector *a*2 become parallel; [10 $\bar{1}\bar{4}$ ] represents the projection of *c*-axis [0001] on the (20 $\bar{2}$ 1) plane, where [1 $\bar{2}$ 10] and [10 $\bar{1}\bar{4}$ ] are orthogonal crystal directions. According to previous study, besides inherent valence band level splitting of semipolar (*r*-plane) of InGaN [32, 34, 42, 43], PSS could intensify the unbalanced biaxial stress in the semipolar active region of InGaN and lead to enhanced splitting of valence band levels [44]. Figure 1(c) demonstrates the relative positions of the heavy-hole (HH) band, light-hole (LH) band, and crystal-field split hole (CH) band allowed for polarized spontaneous emission. For wurtzite GaN, light produced with E⊥c* polarization is favorable for the C-LH transition, while light with E//c* polarization is dominant for the C-HH transition [32, 45, 46].

**Figure 2:**
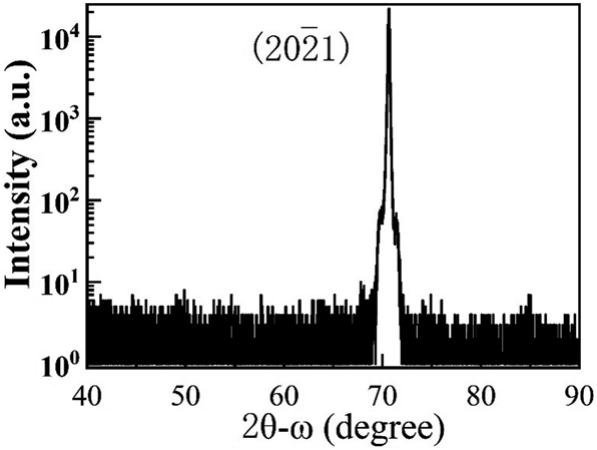
XRD 2*θ*–*ω* scan profile curve of semi-polar (20 $\bar{2}$ 1) InGaN/GaN SQWs.

The crystal quality and dislocation density of the epitaxial film have a crucial impact on the performance of the device. Figure 2 displays the 2*θ*/*ω* scanning curve of GaN(20–21). The diffraction peak is located at 70.7°, with a full width at half maximum (FWHM) of about 516 arcsec. The narrow half-width suggests good crystal quality [35].

**Figure 3:**
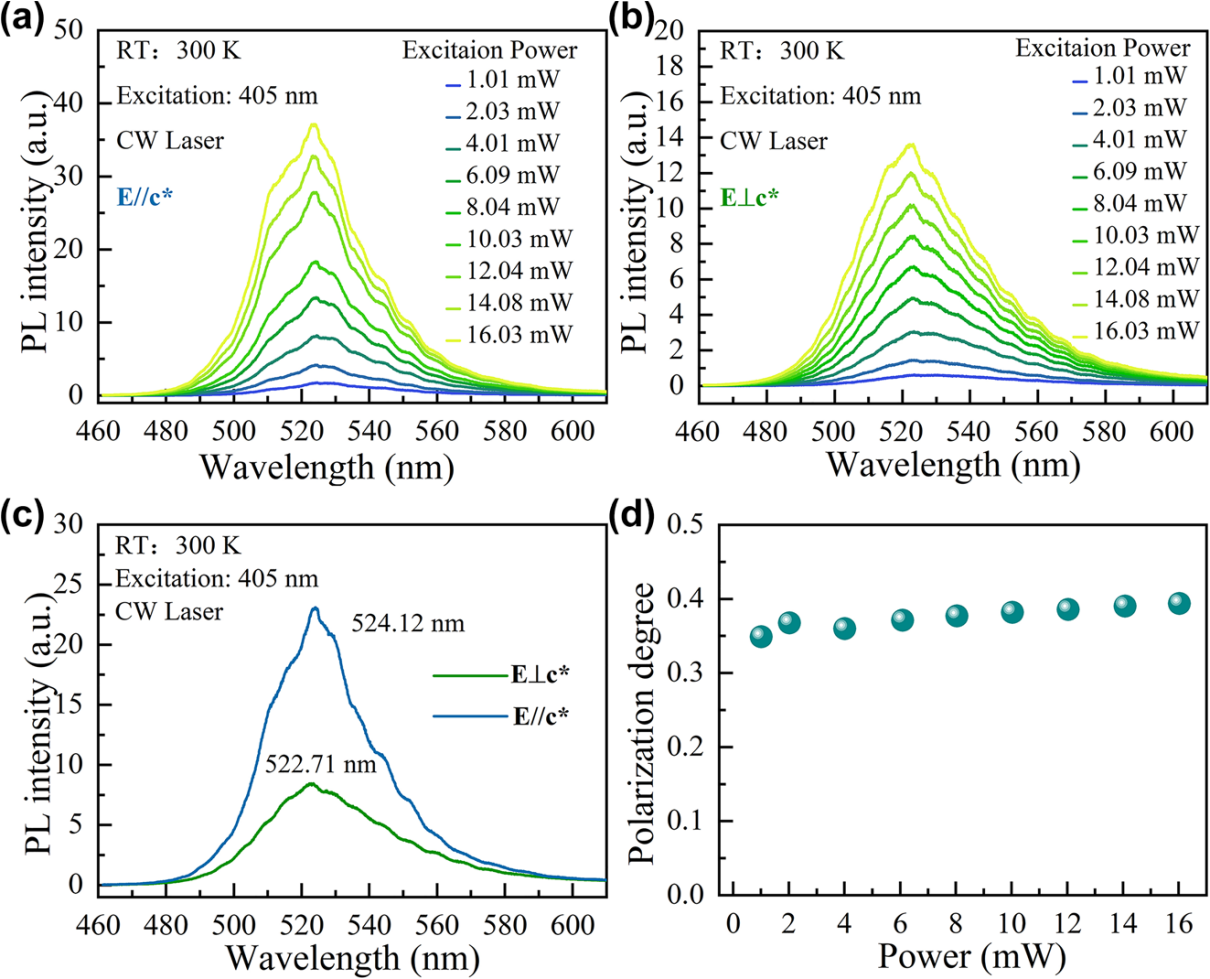
Shows polarization of PL spectra of the epitaxial wafer. (a), (b) PL spectra of the epitaxial wafer with varying excitation power along //c* and ⊥c*, respectively, at room temperature; (c) PL spectra of the epitaxial wafer along //c* and ⊥c* at room temperature subjected to 10 mW excitation power; (d) polarization degree with increasing excitation power.

Figure 3(a) and (b) illustrate the power-dependent PL spectra of the polarizer-filtered epi-wafer along //c* and ⊥c*, respectively. The spectra of both polarization directions show almost no blue-shift with increasing excitation power, reflecting the weak built-in electric field and small QCSE in the semipolar InGaN quantum well. A negligible QCSE improves the overlap of the electron and hole wave functions and enhances the carrier recombination efficiency [47, 48]. Figure 3(c) demonstrates the PL spectra of the wafer in the orthogonal direction under the same excitation energy of 10 mW. The central wavelength with E⊥c* polarization is 522.71 nm, whereas the central wavelength with E//c* polarization is 524.12 nm such that the intensity of the first is weaker. The polarization of the spontaneous emission is in accordance with the splitting valence band of the semipolar InGaN quantum well shown in Figure 1(c). The degree of polarization (*P*) can be evaluated via Eq. (1).

(1) $$P=\frac{I_{\max}-I_{\min}}{I_{\max}+I_{\min}}$$

where *I*_max_ and *I*_min_ in order denote the strongest and weakest spectra. Figure 3(d) presents the degree of polarization of the PL spectrum of the epi-wafer with growing excitation power. The plotted data display that the polarization degree is independent of the excitation and is about 40 %.

**Figure 4:**
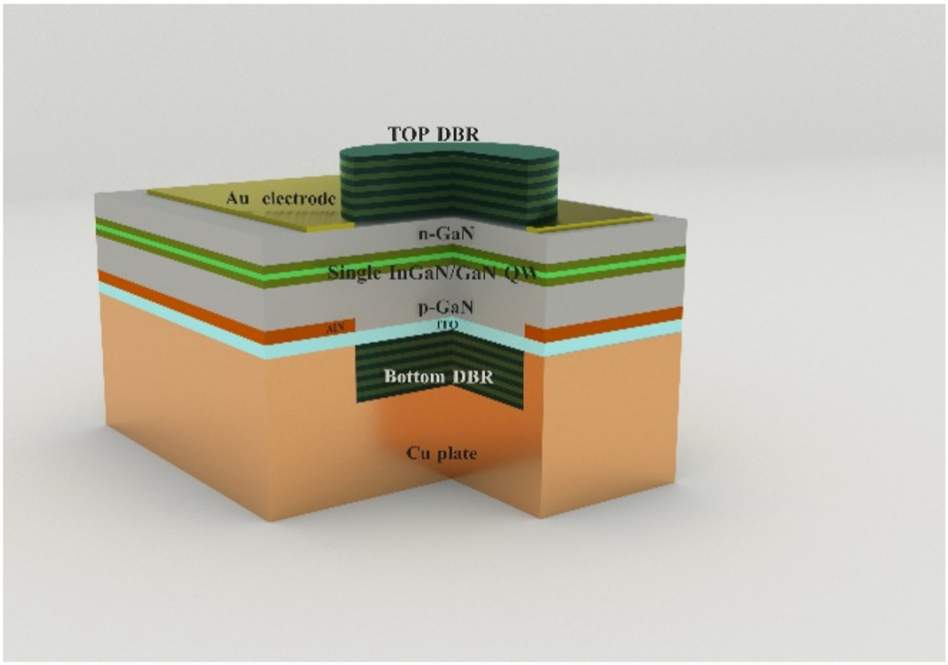
Diagrammatic cross-section of the MCLED.

Using such a wafer, we fabricated (20 $\bar{2}$ 1) InGaN microcavity light-emitting diodes with a double dielectric DBR structure. Figure 4 displays a cross-section of MCLED. In addition to the normal LED structure, the featured microcavity LED structure possesses a vertical resonant cavity composed of top and bottom DBRs.

**Figure 5:**
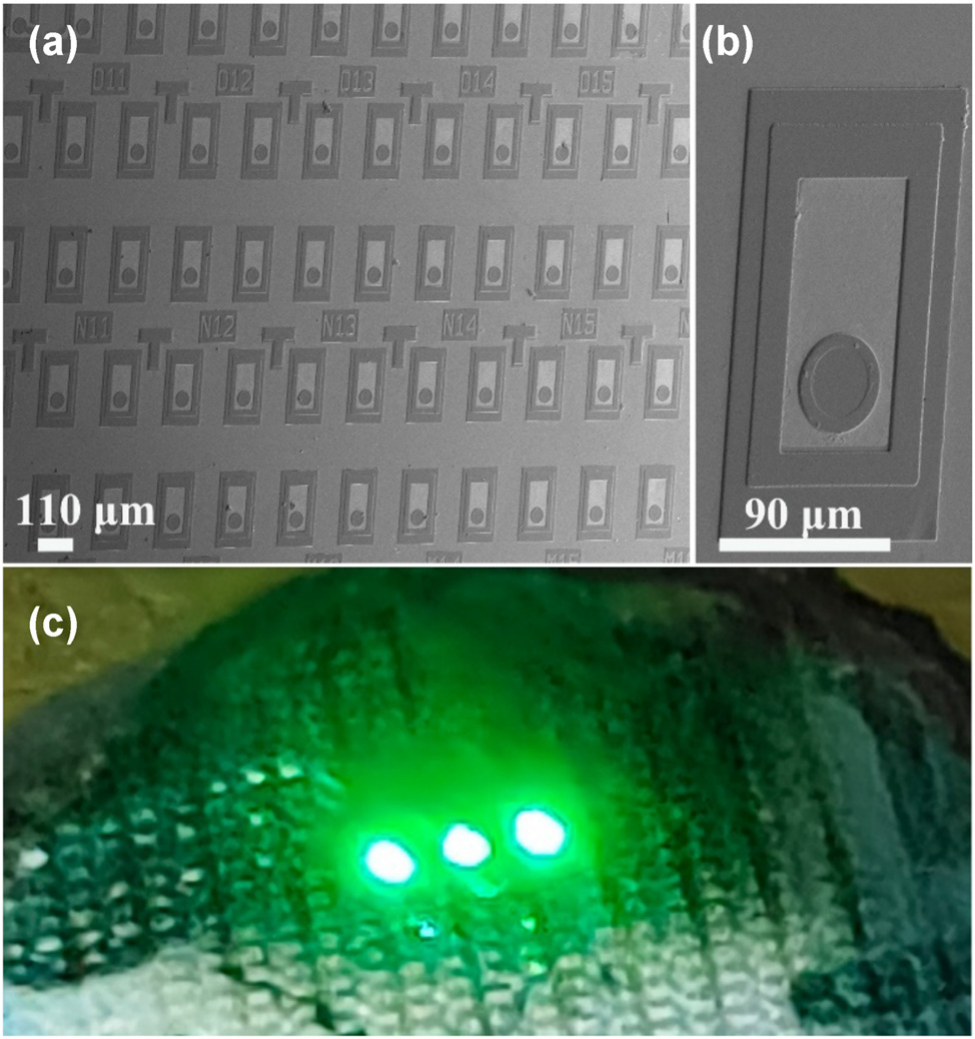
Exhibits outline of MCLEDs. (a) SEM image of the array of MCLEDs on the Cu plate, (b) an SEM-based image of a single MCLED, (c) photograph of three devices in parallel by wire banding in the presence of 3.0 V.

Figure 5(a) and (b) illustrate the SEM images of the MCLED array and a single device. MCLED mesa size is 90 µm by 160 µm. Figure 5(c) shows an actual photo of three devices in parallel by wire banding in the presence of 3.0 V. The luminescence of the three devices was the same, and the polarization of all devices in the array is unified. The small size and highly integrated polarized light source are indispensable for application in wearable devices such as augmented reality (AR) glasses and virtual reality (VR) glasses.

**Figure 6:**
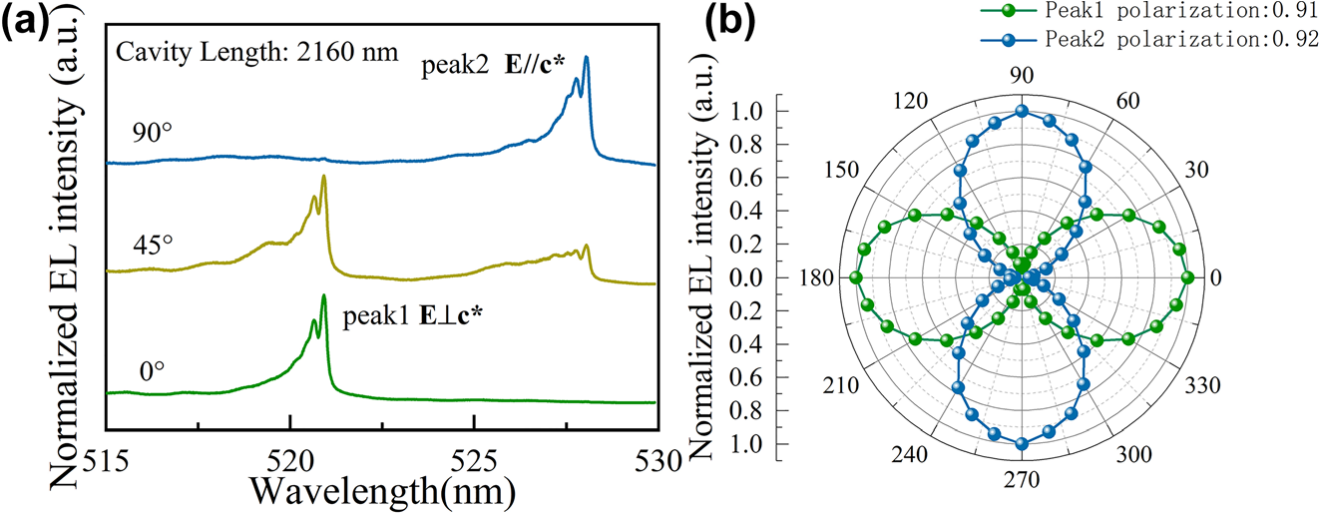
Demonstrates polarization of EL spectra of the MCLED. (a) EL spectra at 0.5 mA of various polarizer angles; (b) normalized EL intensities from (a).

Figure 6(a) illustrates the EL spectra of the semipolar MCLED subjected to various polarizer angles. The two peaks emitted at 520.9 and 528.0 nm could be switched by rotating the polarizer, and the polarization direction is orthogonal. Besides, due to the smaller refractive index of AlN, the current confinement aperture can also confine the optical modes laterally inside the aperture. Therefore, both the two EL peaks show multi-peak structure, which corresponds to the fundamental mode and higher-order transverse modes. The phenomenon can always be found in microcavity devices with lateral optical guide structure [47]. Additionally, the fundamental mode FWHM is 0.2 nm, which is similar to the FWHM of a normal laser. Figure 6(b) demonstrates the polarization-dependent intensity of the two peaks measured by rotating the polarizer from 0° to 360°. For each individual peak, the polarization degree is almost 100 %.

**Figure 7:**
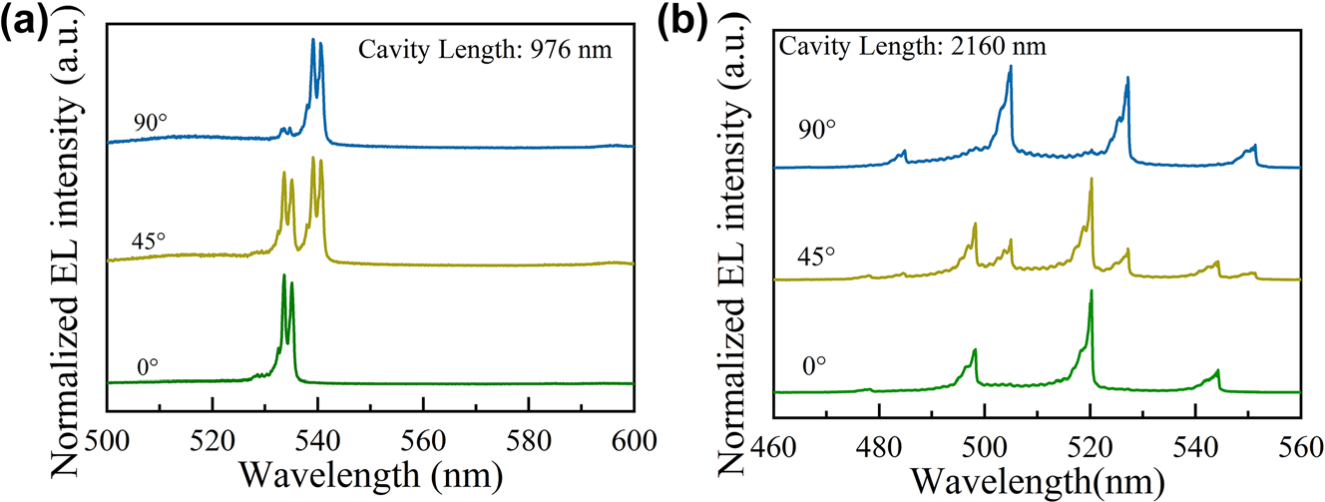
Demonstrates single-mode and multi-mode EL spectra from MCLEDs with different cavity length. (a) Single-mode EL spectra obtained from MCLED with a short resonate cavity, 976 nm, (b) multi-mode EL spectra extracted from MCLED with a long resonate cavity, 2160 nm.


Single-mode light sources such as lasers are commonly exploited in long-distance fiber optic communications to lessen signal interference; while multi-mode light sources are of great vital to enhance data transmission capacity. To meet different applications, we subjectively modulated the cavity length of the MCLED. Figure 7(a) illustrates a single-longitudinal mode of the EL spectrum of an MCLED with a resonant cavity length of 976 nm. Figure 7(b) illustrates a multi-longitudinal mode EL spectrum of another MCLED with a resonant cavity length of 2160 nm. Obviously, in the wavelength range from 500 nm to 600 nm, the spectra of the former device exhibit only one pair of orthogonal linearly polarized longitudinal modes at 535.13 nm and 540.72 nm. On the other hand, four pairs of orthogonal linearly polarized longitudinal modes are detectable in the latter device within the wavelength interval of 460 nm–560 nm. The main reason is that the separation between adjacent longitudinal modes strongly relies on the length of the resonant cavity, formulated in Eq. (2), where *L* denotes the length of the resonant cavity, *λ* represents the wavelength of the mode, Δ*λ* is the separation between adjacent longitudinal modes, *n* signifies the internal refractive index of the resonant cavity. Finally, the term [1−(*λ*/*n*)(d*n*/d*λ*)] is essentially related to the dispersion of the refractive index of the material.

(2) $$\Delta \lambda =\frac{\lambda^{2}}{2nL\left[1-\left(\lambda /n\right)\left(\text{d}n/\text{d}\lambda \right)\right]}$$

**Figure 8:**
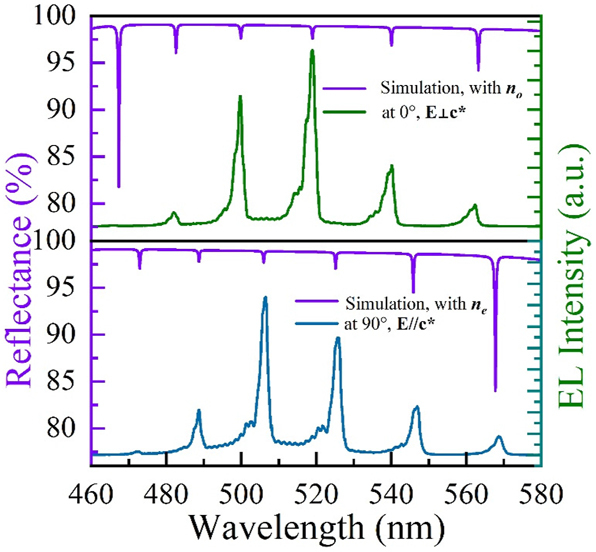
Comparison of the simulated reflectance spectra and EL spectra of the MCLED at various polarization angles.

The simulated reflection spectrum and the corresponding EL spectrum of MCLED with electric fields ⊥c* and //c* are presented in Figure 8. The values of *n*_o_ and *n*_e_ are listed in Table 1 and utilized in simulation calculations, where no represents the refractive index of ordinary light (o Light), and n_e_ denotes the refractive index of extraordinary light (e light) in a birefringent material. From the simulated reflection spectrum, the cavity mode at about 482.05 nm, 499.72 nm, 518.98 nm, 540.20 nm, and 562.43 nm with the electric field along the ⊥c* direction, and 488.80 nm, 506.47 nm, 525.91 nm, 546.95 nm, and 568.80 nm along //c* direction, respectively. These values are precisely match with those of the EL spectra of different polarizations.

**Table 1:** The values of *n*_o_ and *n*_e_ for various materials.

Material	*n*_o_	*n*_e_
GaN	2.408	2.435
InGaN	2.724	2.771
AlGaN	2.305	2.435

**Figure 9:**
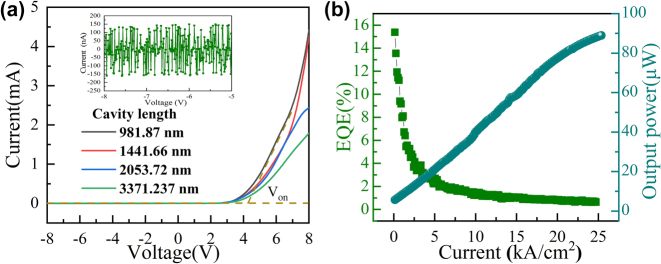
Demonstrates the optoelectronic performance of each devices. (a) I–V curve of the MCLED, where the inset signifies the reverse leakage current; (b) light output power at various currents.

Finally, we focused on the optoelectronic performance of each device. Figure 9(a) presents the I–V curve of the devices with different cavity lengths, and the inset represents the reverse leakage current. For device with cavity length of 981.87 nm, the forward turn-on voltage was about 4.2 V, and the reverse leakage current was around 50 nA in the presence of −8 V. The turn-on voltage and resistance of the device gradually increase with a larger cavity length. This is because the thickness of n-GaN determines the cavity length of the device. Longer cavity means longer electron transport distance, which increases the resistance of the device. Figure 9(b) demonstrates the light output intensity and the external quantum efficiency depended on injection current of the MCLED. When the injection current was about 25.0 kA/cm^2^ (corresponding to a current of 20.0 mA), the maximum output power was 90 μW. Additionally, the external quantum efficiency is about 15 % under 0.16 kA/cm^2^.

## 3 Discussion

**Figure 10:**
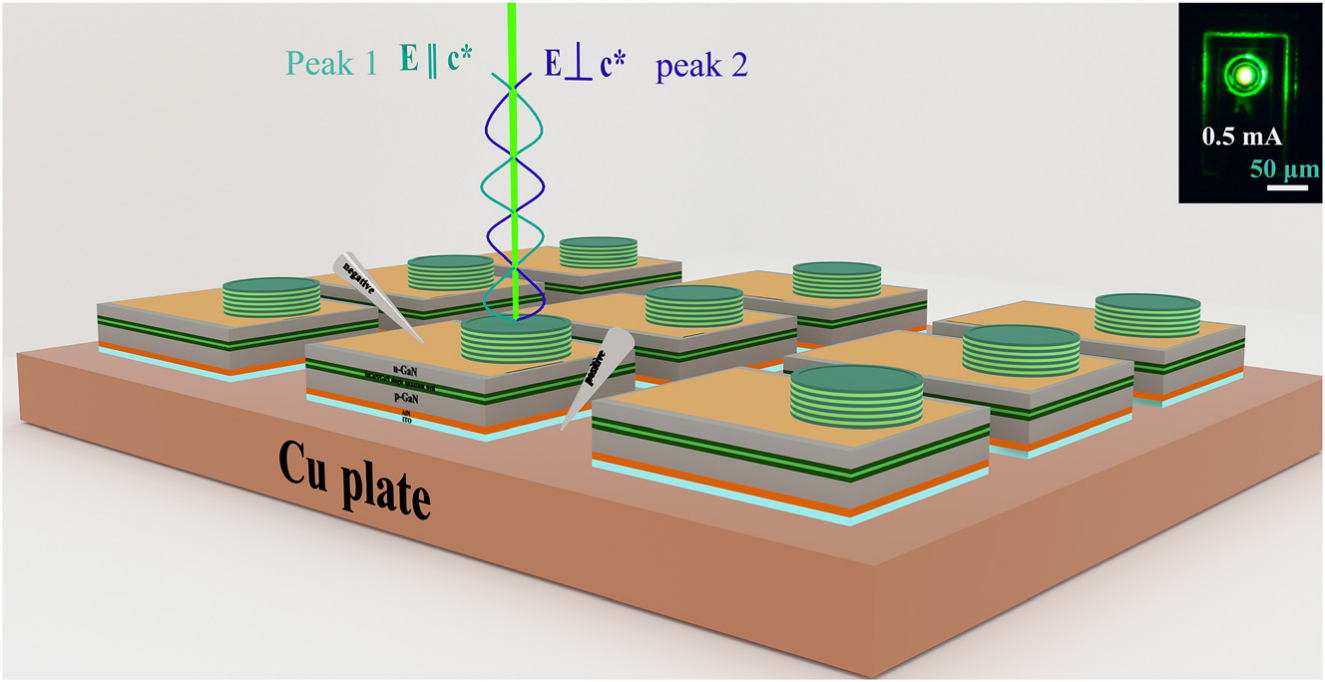
Schematic representation of a pair of orthogonal linearly polarized light from MCLED, where the presented inset is a real photograph of MCLED lighting in the presence of 0.5 mA.

As depicted in Figure 10, we can rationally claim that peak 1 and peak 2 are mutually orthogonal linearly polarized light. Besides, the orthogonal direction corresponds to the polarization direction of the PL spectrum of the epitaxial wafer. Furthermore, the insert in Figure 9 illustrates the luminescence of the device at an injection current of 0.5 mA. The brightest spot was in the center of the confinement aperture. The polarization characteristics of the EL spectra are similar to those of the Zeeman dual-frequency orthogonal laser spectra. But MCLED’s incoherent light is capable of destroying the interference points. In addition, such polarization characteristics can be applied to polarization division multiplexing to increase the data transfer rate and provide another degree of freedom. Furthermore, separate spectra are able to avoid interference between individual sensors in wavelength division multiplexing, which improves the quality of data transmission. Moreover, high polarization and narrow spectra are useful for improving color purity in 3D displays.

It has been shown the PL of a semipolar InGaN/GaN single quantum well (20 $\bar{2}$ 1) exhibits stable linearly polarized spontaneous emission, owing to valence band level splitting. Additionally, by employing a proven fabrication process, we could successfully fabricate (20 $\bar{2}$ 1) semipolar InGaN/GaN MCLEDs. Due to the resonance cavity effect and birefringence of semipolar GaN, the devices are able to simultaneously emit linearly polarized (E//c* and E⊥c*) light with a narrow FWHM of 0.2 nm. Further, MCLEDs are capable of operating in the presence of single-mode or multi-mode by intentionally changing the resonance cavity length. Through exploiting the same device structure and material system with various In or Al contents, novel MCLEDs can be suitably fabricated that simultaneously emit orthogonal and linear polarized red, blue, and ultraviolet lights, which will be promising for various novel applications such as generation of orthogonally polarized white light.

## 4 Methods

### 4.1 The experimental setup to measure the polarization of emission

**Figure 11:**
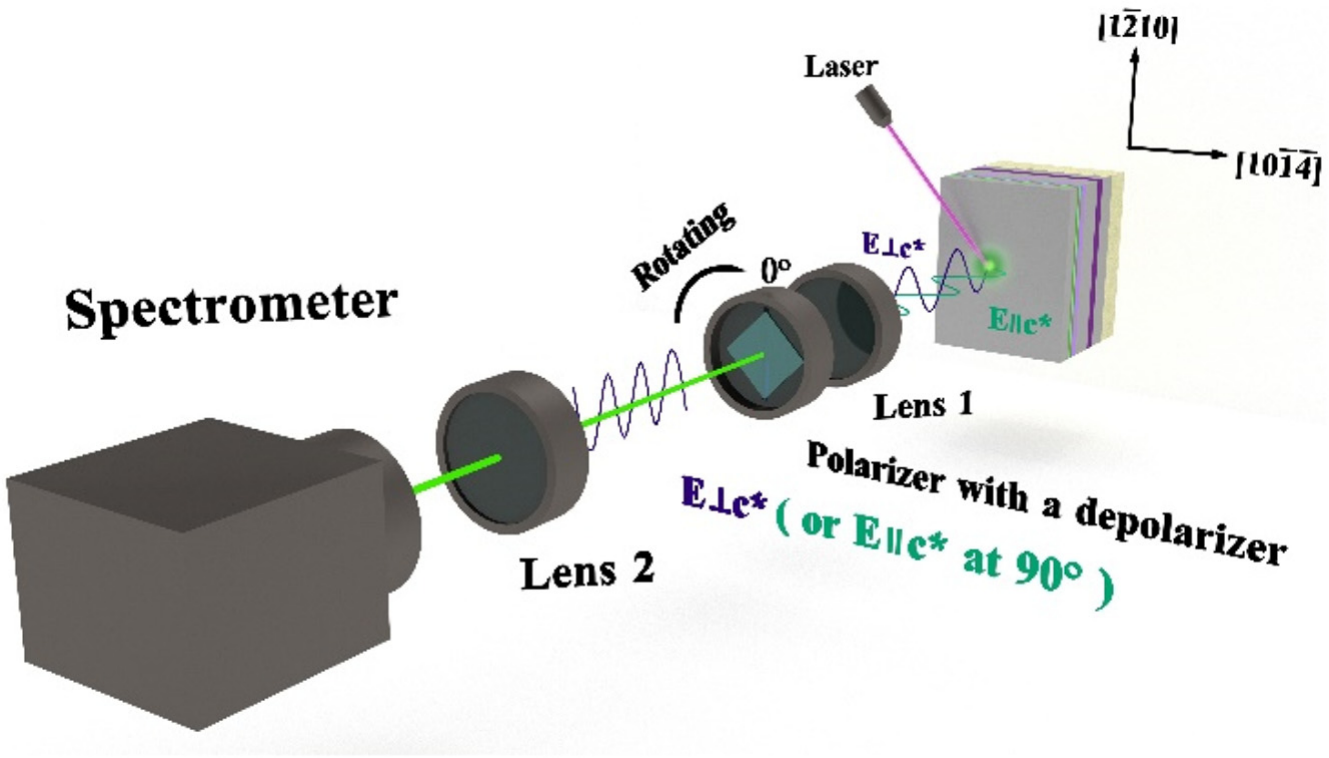
Schematic illustration of the experimental setup to measure the polarization of emission spectra.

A 405 nm laser diode is employed as the excitation source to measure the PL spectra of the wafer at room temperature, where the measurement system has been presented in Figure 11. The spontaneous emission of the wafer is collected by lens 1, and the polarization direction is suitably resolved by a polarizer. The polarizer silt at 0° is parallel to the direction of PSS grooves and terraces (⊥c*). Note that the depolarizer is placed between the polarizer and the spectrometer to eliminate possible polarization selectivity of the spectrometer grating.

### 4.2 The process of device fabrication

**Figure 12:**
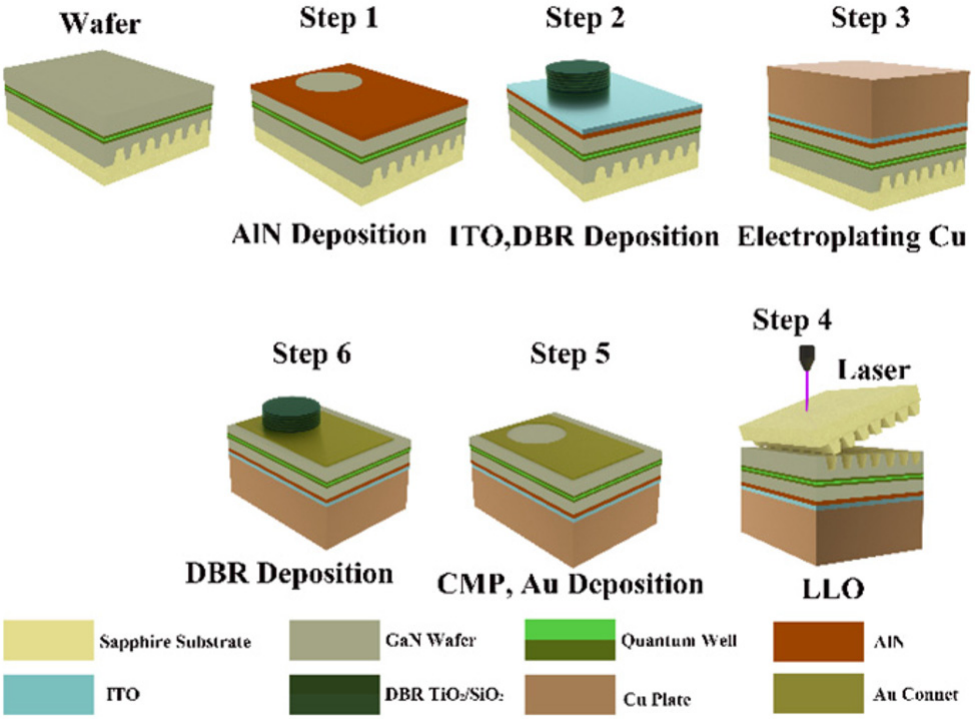
Schematic representation of the device fabrication process.

**Figure 13:**
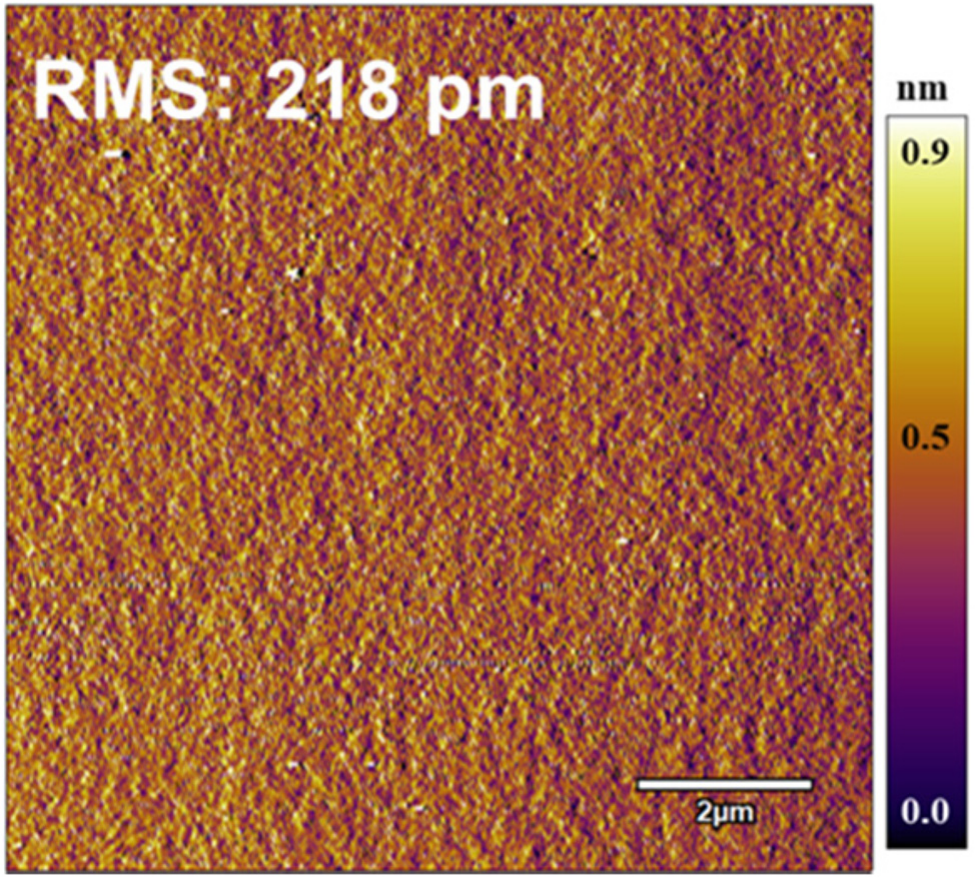
After CMP, the surface of n-GaN via AFM.

Figure 12 demonstrates the flowchart of the device manufacturing process. First, a mesa with a step height of 30 nm was etched on the p-GaN surface through a lithography process and inductively coupled plasma (ICP) etching. And then, the other area was filled by a 30 nm thick AlN film through magnetron sputtering, which acted as a current confinement layer. The diameter of the AlN encapsulation layer surrounded by p-GaN is 10 μm, which acts as a light-emitting aperture. To enhance current spreading, ITO was deposited on the wafer surface. After that, a bottom DBR structures were deposited at room temperature using ion-assisted electron gun evaporation. The bottom DBR included 12 pairs of TiO_2_/SiO_2_. In order to improve the reflectivity, the optical thickness of each layer of TiO_2_/SiO_2_ was 1/4*λ* (*λ* is the central wavelength), where the thickness of each layer of TiO_2_ and SiO_2_ were 53.22 nm and 87.94 nm, respectively. To realize the dual structure of DBR, film transfer was performed by electroplating copper supporting plate and laser lift-off (LLO). Since high surface roughness is capable of enhancing optical scattering losses, chemical mechanical polishing (CMP) is adopted to arrive at a smooth n-GaN surface after LLO. After CMP, the final surface would be smooth with a roughness of 0.218 nm at a size of 10 × 10 μm^2^ size scanned by an atomic force microscope (AFM), as illustrated in Figure 13. At last, device mesas were separated by ICP etching, and Cr/Au negative electrode and a top DBR with 8 pairs of TiO_2_/SiO_2_ were deposited. The copper substrate and AlN confinement aperture with high thermal conductivity l are crucial for improving the thermal dissipation of the MCLED [49].
